# Genetic interaction of *DISC1* and *Neuroligin* in regulation of glutamatergic synaptogenesis

**DOI:** 10.3389/fnins.2026.1832180

**Published:** 2026-05-28

**Authors:** Takato Honda, Himani Pandey, Akira Sawa, Katsuo Furukubo-Tokunaga

**Affiliations:** 1 The Picower Institute for Learning and Memory, Department of Brain and Cognitive Sciences, Massachusetts Institute of Technology (MIT), Cambridge, MA, United States; 2 Stanley Center for Psychiatric Research, Broad Institute of MIT and Harvard, Cambridge, MA, United States; 3 Department of Anesthesia, Critical Care, and Pain Medicine, Massachusetts General Hospital and Harvard Medical School, Boston, MA, United States; 4 Life and Environmental Sciences, University of Tsukuba, Tsukuba, Japan; 5 Department of Biotechnology, Mahatma Gandhi Central University, Motihari, Bihar, India; 6 Departments of Psychiatry, Neuroscience, Mental Health, Pharmacology, Biomedical Engineering, and Genetic Medicine, Johns Hopkins University School of Medicine and Bloomberg School of Public Health, Johns Hopkins Medicine, Baltimore, MD, United States

**Keywords:** *DISC1*, genetics, molecular and biological psychiatry, *Neuroligin*, schizophrenia, synapse

## Abstract

Synaptic development and functionality have been considered as fundamental mechanisms underlying a range of neuropsychiatric disorders, including schizophrenia. Emerging evidence from biological and clinical studies implicates mutations in the *Disrupted-in-Schizophrenia-1 (DISC1)* gene in the pathogenesis of schizophrenia. In this study, genetic interactions among a select set of risk factor genes are examined using the unique model system: neuromuscular junctions (NMJs) in *Drosophila*. We found that *dnlg1*, the *Drosophila* homolog of the human *Neuroligin-1*, which encodes the NLGN1 protein, genetically interacts with *DISC1* in synaptic development. We show that *DISC1* overexpression in the *dnlg1^null^* heterozygous background causes synaptic alterations at the NMJs that are distinct from those in the wild-type background. Loss of *dnlg1* enhanced the *DISC1* overexpression phenotype in synaptic formation, strongly suppressing the formation of synaptic boutons. These results thus suggest an intriguing converging mechanism regulated by *DISC1* and *Neuroligin* in the developing glutamatergic synapses.

## Introduction

Schizophrenia is a debilitating psychiatric disorder affecting approximately 1% of the global population (American Psychiatric Association, 2022). While the precise molecular and pathological mechanisms underlying schizophrenia remain elusive, findings from genome-wide association studies and familial analyses have identified multiple genetic risk factors contributing to its etiology. To date, a growing number of susceptibility loci have been reported (Luo et al., 2024; Pardiñas et al., 2018; Schizophrenia Working Group of the Psychiatric Genomics Consortium, 2014). These investigations further reveal that several schizophrenia-associated genetic loci are also implicated in other psychiatric conditions, such as bipolar disorder, autism spectrum disorder, and intellectual disability (McCarthy et al., 2014). A significant proportion of these shared loci encode synaptic proteins, suggesting a convergence of genetic risk on pathways that govern synaptic development and plasticity (Hall et al., 2015). Collectively, these results reinforce the synaptic hypothesis, proposing that impaired neuronal connectivity and signaling may be central to the neuropathology observed in affected individuals (Moghaddam and Javitt, 2012).

**Figure 1:**
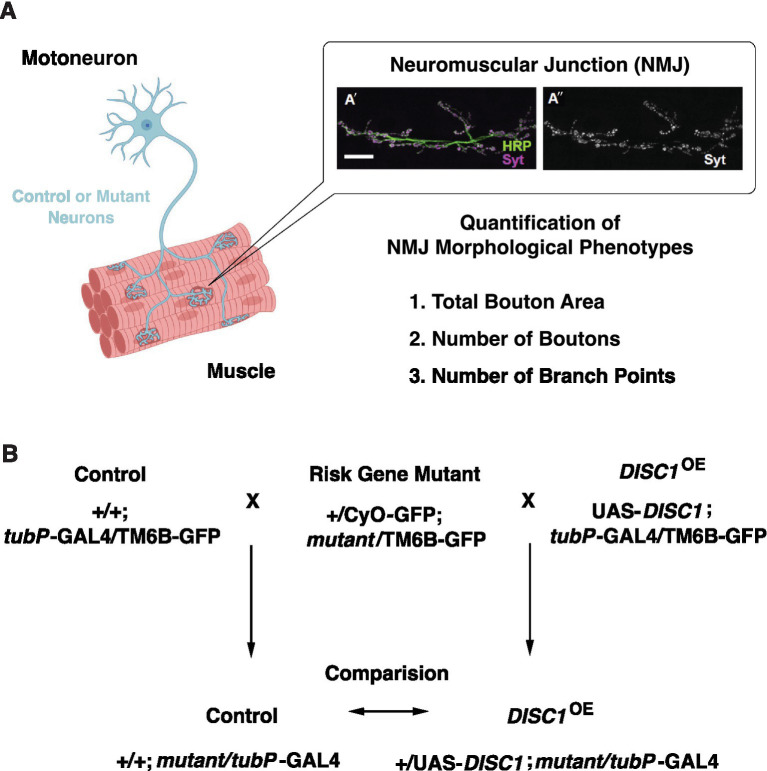
Quantifications of NMJ morphological phenotypes and screening of *DISC1*-interacting genes. **(A)** A schematic presentation and a confocal image of the *Drosophila* larval NMJs. The larval NMJs display stereotypic synaptic connections between the identifiable presynaptic motoneuron and the specific postsynaptic muscles. NMJs on the muscle 6/7 in the second abdominal segment were stained with anti-HRP (green) and anti-SYT (magenta) antibodies. Scale bar, 20 μm. HRP: horseradish peroxidase protein. Syt: synaptotagmin. Quantification of the NMJ morphological phenotypes is based on (1) the total bouton area (μm^2^), (2) the number of boutons, and (3) the number of branch points. **(B)** Genetic screening of *DISC1*-interacting genes. Mutant flies (+*/CyO-GFP*; *mutation*/TM6B-GFP) of the fruit fly homolog for a schizophrenia risk factor gene (*dnlg-1* in this study) are crossed with the control (+/+; *tubP*-*GAL4*/*TM6B-GFP*) or the *DISC1* ^OE^ (*UAS-DISC1*; *tubP-GAL4*/*TM6B-GFP*) flies. The phenotypes of the larval NMJs between the control (+*/*+*; mutation/tubP-GAL4*) and *DISC1* ^OE^ (+*/UAS-DISC1; mutation/tubP-GAL4*) animals were compared.

The larval neuromuscular junction (NMJ) of *Drosophila melanogaster* exhibits several key features analogous to those of excitatory synapses in the vertebrate brain (Charng et al., 2014). At the fly NMJ, glutamate serves as the principal neurotransmitter, and the ionotropic glutamate receptors present are homologous to those found in humans (Collins and DiAntonio, 2007). Additionally, akin to vertebrate central synapses, synapses at the *Drosophila* NMJ display dynamic plasticity, characterized by a regulated pattern of bouton formation and elimination during development and synaptic remodeling (Bayat et al., 2011). The stereotyped synaptic architecture of the fly NMJ, with clearly defined presynaptic motoneurons and postsynaptic muscle fibers, renders it a powerful model for studying the molecular and genetic basis of synapse formation and function (Figure 1A) (Charng et al., 2014).

To investigate the interactions among various psychiatric risk genes involved in synaptogenesis, we introduced the human *DISC1* gene into *Drosophila*. A balanced chromosomal translocation disrupting the *DISC1* locus was first identified in a large Scottish pedigree affected by multiple psychiatric conditions, including schizophrenia, major depressive disorder, bipolar disorder, and autism spectrum disorder (Brandon and Sawa, 2011; Bradshaw and Porteous, 2012). In prior work, we demonstrated that overexpression of *DISC1* (*DISC1^OE^*) leads to structural abnormalities at the larval neuromuscular junction, specifically reducing the overall bouton area (Furukubo-Tokunaga et al., 2016). Based on this finding, we have genetically screened other schizophrenia risk factor genes for interactions with *DISC1* in the developing glutamatergic synapses and identified potential interacting genes (Figure 1B and Supplementary Table S2). In the present study, we show that loss of *dnlg1*, the *Drosophila* homolog of human *Nuroligin-1* (Scheiffele et al., 2000; Banovic et al., 2010), enhances the *DISC1^OE^* phenotype in synaptogenesis, strongly suppressing the formation of synapse boutons. These results suggest an intriguing converging mechanism controlled by *Neuroligin* and *DISC1* in the developing glutamatergic synapses.

## Methods

### Subjects

As the *Drosophila* standard stock, a *white* (*w*) stock outcrossed with *Canton S* 10 times [*w (CS10)*] was used in the present study as controls. The details of the construction of transgenic flies carrying the *UAS-DISC1* transgene were described previously (Furukubo-Tokunaga et al., 2016). The fly stocks were outcrossed to *w (CS10)* at least 5 times to ensure a homogeneous genetic background. *dnlg1* null mutant (*dnlg1^MI03763^*) (Banovic et al., 2010; Nagarkar-Jaiswal et al., 2015) and *GAL4* driver (*tubP-GAL4*) (O'Donnell et al., 1994) were obtained from the Bloomington Stock Center (Bloomington, IN, USA). All stocks were raised at 25 °C on a standard fly food.

### Genetic screening

The screening scheme is described in Figure 1B and previously (Furukubo-Tokunaga et al., 2016; Pandey et al., 2017; Honda et al., 2024). First, the mutant lines carrying target risk genes were balanced with a double balancer stock (*w/w; Sp/CyO Act-GFP; Pr Dr/TM6B ubi-GFP*) for this genetic screening. Then, the resulting progeny carrying a mutation were crossed either with control (*w; +; tubP-GAL4/TM6B ubi-GFP*) or with *DISC1^OE^* (*w; UAS-DISC1(CS10)6-6(II); tubP-GAL4/ TM6B ubi-GFP*) flies. Larvae were raised at 25 °C, and non-GFP expressing flies were selected to dissect and quantify NMJ phenotypes without being blinded to genotypes at 116–120 h after egg collection. Both male and female flies are randomly used in this study. Our genetic screening is summarized in Supplementary Table S2, listing the human risk genes and their homologous *Drosophila* mutants that we have been examining the interactions with *DISC1*, along with our previously reported studies.

### Immunohistochemistry

Immunological staining was performed based on our previous methods (Kurusu et al., 2002). In this study, the following antibodies were used: sheep anti-DISC1 antibody diluted 1:50 (AF6699, R&D systems, Minneapolis, MN, USA), mouse anti-synaptotagmin diluted 1:2 (3H2 2D7, Developmental Studies Hybridoma Bank (DSHB), University of Iowa, IA, USA), anti-horseradish peroxidase protein (HRP) conjugated with fluorescein-isothiocyanate diluted 1:50 (Jackson ImmunoResearch, West Grove, PA, USA), and Alexa-conjugated secondary antibodies diluted 1:1000 (Molecular Probes, Eugene, OR, USA). We captured the confocal images using a Zeiss LSM510 and LSM710 microscope.

### Quantification of NMJ structure

**Figure 2:**
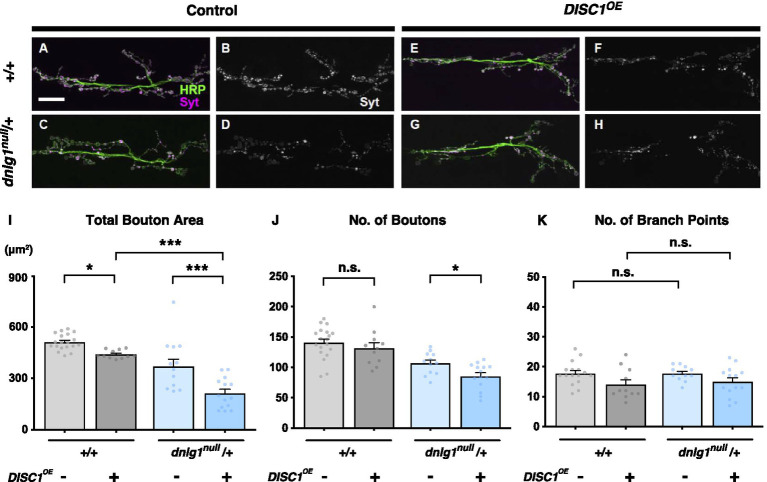
*DISC1* interacts with *dnlg1* in synaptogenesis. **(A–H)** Representative confocal images of NMJs with (+) or without (−) *DISC1* expression (*DISC1^OE^*) in Control *w (CS10)* +/+ or *dnlg1^null^*/+ backgrounds. NMJs on the muscle 6/7 in the second abdominal segment were immunostained with anti-HRP (green) and anti-SYT (magenta) antibodies. Scale bar, 20 μm. HRP: horseradish peroxidase protein. Syt: synaptotagmin. **(I–K)** Quantification of the NMJ morphology of the total bouton area (μm^2^) **(I)**, the number of boutons **(J)**, and the number of branch points **(K)** in heterozygous *dnlg1^null^* animals with (+) or without (−) *DISC1* overexpression (*DISC1^OE^*). Two-way ANOVA followed by Holm-Sidak’s multiple comparisons. n.s. not significant. *p < 0.05, ***p < 0.0001. Data are presented as the means with SEM. The full table of statistical results for the displayed figure is in Tables 1–3 (Two-way ANOVA and multiple comparisons) and Supplementary Table S1 (Normality test), including the number of biological replicates and the statistical values for all tests.

To quantify synaptic phenotypes, we used the larval longitudinal muscles 6/7 in the abdominal hemisegment A2 (Figures 1A, 2) using Image-J (http://rsb.info.nih.gov/ij/), consistent with our previously described methods (Furukubo-Tokunaga et al., 2016; Pandey et al., 2017; Honda et al., 2024). For NMJ structural quantification, we used anti-HRP and anti-synaptotagmin antibodies to label neuronal terminals and synaptic boutons, respectively (see ‘Immunohistochemistry’ for antibody specifications). The total bouton area (μm^2^) and the number of boutons were determined based on the detected areas with the signals of anti-synaptotagmin immunoreactivity (Figure 1A). The number of branch points was evaluated based on the axonal branch structures labeled with the pan-neuronal maker, anti-HRP immunoreactivity (Figure 1A).

### Statistics

Statistical analysis was performed using GraphPad Prism (Version 11.0, GraphPad Software, MA, USA) and OriginPro (Version 10.2, OriginLab Corporation, MA, USA). All data were subjected to D’Agostino–Pearson (Omnibus K2) normality test and Shapiro–Wilk normality test for Gaussian distribution and variance. All datasets in the present study showed a Gaussian distribution (passed normality tests; see Supplementary Table S1 for all the statistical results). Thus, we performed a parametric test, a Two-way ANOVA, followed by Holm-Sidak’s multiple comparisons only for factor groups with a significant (*p* < 0.05) main effect by a factor (Figure 2 and see Tables 1–3 for all statistical results). Significance levels in the figures are represented as *p* < 0.05 (*), *p* < 0.01 (**), *p* < 0.0001 (***). Data are presented as the mean with the standard error of the mean (SEM).

**Table 1:** Analysis for Figure 2I total bouton area: two-way ANOVA and multiple comparisons.

**Table analyzed**	**Two-way ANOVA:** **Figure 2I** **Total bouton area**				
Two-way ANOVA	Ordinary				
Alpha	0.05				
Source of Variation	% of total variation	*p* value	*P* value summary	Significant?	
Interaction	2.89	0.0431	*	Yes	
dnlg1 null (−) or (+)	17.09	<0.0001	***	Yes	
DISC1 OE (−) or (+)	36.9	<0.0001	***	Yes	
ANOVA table	SS (Type III)	DF	MS	F (DFn, DFd)	*P*-value
Interaction	33,240	1	33,240	*F* (1, 52) = 4.300	*P* = 0.0431
dnlg1 null (−) or (+)	196,525	1	196,525	*F* (1, 52) = 25.43	*P* < 0.0001
DISC1 OE (−) or (+)	424,361	1	424,361	*F* (1, 52) = 54.90	*P* < 0.0001
Residual	401,937	52	7,730		
Total	1,149,993	55			
Difference between column means					
Predicted (LS) mean of +/+	475.6				
Predicted (LS) mean of dnlg1null/+	297.7				
Difference between predicted means	177.9				
SE of difference	24.01				
95% CI of difference	129.7 to 226.1				
Difference between row means					
Predicted (LS) mean of DISC1OE (−)	447.2				
Predicted (LS) mean of DISC1OE (+)	326.1				
Difference between predicted means	121.1				
SE of difference	24.01				
95% CI of difference	72.88 to 169.2				
Interaction CI					
Mean diff, A1 – B1	128.1				
Mean diff, A2 – B2	227.7				
(A1 – B1) – (A2 – B2)	−99.58				
95% CI of difference	−195.9 to −3.221				
(B1 – A1) – (B2 – A2)	99.58				
95% CI of difference	3.221 to 195.9				
Data summary					
Number of columns (DISC1 OE (−) or (+))	2				
Number of rows (dnlg1 null (−) or (+))	2				
Number of values	56				

Multiple comparisons: Figure 2I Total bouton area								
Number of families	1							
Number of comparisons per family	3							
Alpha	0.05							
Holm-Šídák’s multiple comparisons test	Predicted (LS) Mean diff.	Below threshold?	Summary	Adjusted *P-*value				
DISC1OE (−):+/+ vs. DISC1OE (+):+/+	71.27	Yes	*	0.0371				
DISC1OE (−):dnlg1null/+ vs. DISC1OE (+):dnlg1null/+	170.9	Yes	***	<0.0001				
DISC1OE (+):+/+ vs. DISC1OE (+):dnlg1null/+	227.7	Yes	***	<0.0001				
Test details	Predicted (LS) Mean 1	Predicted (LS) Mean 2	Predicted (LS) Mean diff.	SE of diff.	N1	N2	*t*	DF
DISC1OE (−):+/+ vs. DISC1OE (+):+/+	511.3	440	71.27	33.31	19	11	2.14	52
DISC1OE (−):dnlg1null/+ vs. DISC1OE (+):dnlg1null/+	383.1	212.3	170.9	34.59	12	14	4.94	52
DISC1OE (+):+/+ vs. DISC1OE (+):dnlg1null/+	440	212.3	227.7	35.42	11	14	6.428	52

**Table 2:** Analysis for Figure 2J number of boutons: two-way ANOVA and multiple comparisons.

**Table analyzed**	**Two-way ANOVA:** Figure 2J **Number of boutons**				
Two-way ANOVA	Ordinary				
Alpha	0.05				
Source of Variation	% of total variation	*P*-value	*P*-value summary	Significant?	
Interaction	0.437	0.5025	ns	No	
dnlg1 null (−) or (+)	7.416	0.0075	**	Yes	
DISC1 OE (−) or (+)	35.84	<0.0001	***	Yes	
ANOVA table	SS (Type III)	DF	MS	*F*(DFn, DFd)	*P*-value
Interaction	236	1	236	*F*(1, 52) = 0.4560	*P* = 0.5025
dnlg1 null (−) or (+)	4,005	1	4,005	*F*(1, 52) = 7.739	*P* = 0.0075
DISC1 OE (−) or (+)	19,356	1	19,356	*F*(1, 52) = 37.41	*P* < 0.0001
Residual	26,908	52	517.5		
Total	54,000	55			
Difference between column means					
Predicted (LS) mean of +/+	134.1				
Predicted (LS) mean of dnlg1null/+	96.1				
Difference between predicted means	37.99				
SE of difference	6.212				
95% CI of difference	25.53 to 50.46				
Difference between row means					
Predicted (LS) mean of DISC1OE (−)	123.7				
Predicted (LS) mean of DISC1OE (+)	106.5				
Difference between predicted means	17.28				
SE of difference	6.212				
95% CI of difference	4.816 to 29.75				
Interaction CI					
Mean diff, A1 – B1	33.8				
Mean diff, A2 – B2	42.19				
(A1 – B1) – (A2 – B2)	−8.39				
95% CI of difference	−33.32 to 16.54				
(B1 – A1) – (B2 – A2)	8.39				
95% CI of difference	−16.54 to 33.32				
Data summary					
Number of columns (DISC1 OE (−) or (+))	2				
Number of rows (dnlg1 null (−) or (+))	2				
Number of values	56				

Multiple Comparisons: Figure 2J Number of boutons								
Number of families	1							
Number of comparisons per family	2							
Alpha	0.05							
Holm-Šídák’s multiple comparisons test	Predicted (LS) Mean diff.	Below threshold?	Summary	Adjusted *P-*value				
DISC1OE (−):+/+ vs. DISC1OE (+):+/+	13.09	No	ns	0.135				
DISC1OE (−):dnlg1null/+ vs. DISC1OE (+):dnlg1null/+	21.48	Yes	*	0.0396				
Test details	Predicted (LS) Mean 1	Predicted (LS) Mean 2	Predicted (LS) Mean diff.	SE of diff.	N1	N2	*t*	DF
DISC1OE (−):+/+ vs. DISC1OE (+):+/+	140.6	127.5	13.09	8.618	19	11	1.518	52
DISC1OE (−):dnlg1null/+ vs. DISC1OE (+):dnlg1null/+	106.8	85.36	21.48	8.949	12	14	2.4	52

**Table 3:** Analysis for Figure 2K number of branch points: two-way ANOVA and multiple comparisons.

**Table analyzed**	**Two-way ANOVA:** **Figure 2K** **Number of branch points**				
Two-way ANOVA	Ordinary				
Alpha	0.05				
Source of Variation	% of total variation	*P*-value	*P*-value summary	Significant?	
Interaction	0.222	0.7244	ns	No	
dnlg1 null (−) or (+)	13.28	0.0085	**	Yes	
DISC1 OE (−) or (+)	0.222	0.7244	ns	No	
ANOVA table	SS (Type III)	DF	MS	*F* (DFn, DFd)	*P-*value
Interaction	2.277	1	2.277	*F* (1, 49) = 0.1258	*P* = 0.7244
dnlg1 null (−) or (+)	136.3	1	136.3	*F* (1, 49) = 7.528	*P* = 0.0085
DISC1 OE (−) or (+)	2.277	1	2.277	*F* (1, 49) = 0.1258	*P* = 0.7244
Residual	887.1	49	18.1		
Total	1,026	52			
Difference between column means					
Predicted (LS) mean of +/+	15.92				
Predicted (LS) mean of dnlg1null/+	16.34				
Difference between predicted means	−0.4188				
SE of difference	1.181				
95% CI of difference	−2.792 to 1.954				
Difference between row means					
Predicted (LS) mean of DISC1OE (−)	17.75				
Predicted (LS) mean of DISC1OE (+)	14.51				
Difference between predicted means	3.24				
SE of difference	1.181				
95% CI of difference	0.8669 to 5.614				
Interaction CI					
Mean diff, A1 – B1	4.441E-16				
Mean diff, A2 – B2	−0.8377				
(A1 – B1) – (A2 – B2)	0.8377				
95% CI of difference	−3.909 to 5.584				
(B1 – A1) – (B2 – A2)	−0.8377				
95% CI of difference	−5.584 to 3.909				
Data summary					
Number of columns (DISC1 OE (−) or (+))	2				
Number of rows (dnlg1 null (−) or (+))	2				
Number of values	53				

**Multiple comparisons:** **Figure 2K** **Number of branch points**								
Number of families	1							
Number of comparisons per family	2							
Alpha	0.05							
Holm-Šídák’s multiple comparisons test	Predicted (LS) Mean diff.	Below threshold?	Summary	Adjusted *P*-value				
DISC1OE (−):+/+ vs. DISC1OE (−):dnlg1null/+	0	No	ns	>0.9999				
DISC1OE (+):+/+ vs. DISC1OE (+):dnlg1null/+	−0.8377	No	ns	0.8611				
Test details	Predicted (LS) Mean 1	Predicted (LS) Mean 2	Predicted (LS) Mean diff.	SE of diff.	N1	N2	t	DF
DISC1OE (−):+/+ vs. DISC1OE (−):dnlg1null/+	17.75	17.75	0	1.625	16	12	0	49
DISC1OE (+):+/+ vs. DISC1OE (+):dnlg1null/+	14.09	14.93	−0.8377	1.714	11	14	0.4886	49

## Results

### Genetic screening of *DISC1* interactors in glutamatergic synaptogenesis

To investigate interactions of *DISC1* and other risk factor genes in a genetically tractable model, we overexpressed the human *DISC1* gene in the fruit fly NMJ using the *GAL4*-*UAS* system (Figure 1B). The overexpression of *DISC1* (*DISC1^OE^*) with a ubiquitous *tubP-GAL4* driver caused a significant reduction in the total synaptic bouton area [Figures 2A–I: Two-way ANOVA (*F* (1, 52) = 54.90, *p* < 0.0001), main effect of *DISC1^OE^*] followed Holm-Sidak’s multiple comparisons [+/+; *DISC1^OE^* (−) vs. +/+; *DISC1^OE^* (+), *p* = 0.0371, *t* = 2.140, *df* = 52]. In contrast, the numbers of synaptic boutons was not changed between *DISC1^OE^* (−) and *DISC1^OE^* (+) in the +/+ wildtype background [Figure 2J: Two-way ANOVA (*F* (1, 52) = 37.41, *p* < 0.0001), main effect of *DISC1^OE^*] followed by Holm-Sidak’s multiple comparison [Figure 2J: +/+; *DISC1^OE^* (−) vs. +/+; *DISC1^OE^* (+), *p* = 0.1350, *t* = 1.518, *df* = 52]. *DISC1* overexpression did not affect the numbers of axonal branch points [Figure 2K: Two-way ANOVA (*F* (1, 49) = 0.1258, *p* = 0.7244), main effect of *DISC1^OE^*], which is consistent with the previous studies on *DISC1^OE^* phenotypes (Furukubo-Tokunaga et al., 2016; Pandey et al., 2017; Honda et al., 2024).

To further identify risk factor genes that interact with *DISC1* in synaptogenesis, we set up a genetic crossing between the *DISC1^OE^* animals and the mutants of diverse schizophrenia risk factor genes (Figure 1B and Supplementary Table S2). In the present study, we particularly used the mutations that varied from null to hypomorphic mutants, resulting in a maximum reduction of ~50% in the gene dosage when present in the heterozygous condition used in this genetic screening. They have only a partial impact on synaptic formation when acting independently. To evaluate genetic modifications, we examined the synaptic anatomical phenotypes of NMJs, investigating three morphological parameters (1. Total bouton area, 2. Number of boutons, and 3. Number of branch points) (Figure 1A), consistent with the previously established methods (Furukubo-Tokunaga et al., 2016; Pandey et al., 2017; Honda et al., 2024). We have been analyzing mutations identified on the fly autosomes, which appear to map to the second and third chromosomes (Supplementary Table S2). Despite analyzing a small number of mutations, we identified several genes, such as *dnlg1*, which is the *Drosophila* homolog (Banovic et al., 2010; Mozer and Sandstrom, 2012) of the human *Neuroligin-1* gene (Scheiffele et al., 2000; Südhof, 2008; Krueger and Dean, 2012; Bemben et al., 2015).

### *DISC1* genetically interacts with *dnlg1* in synaptogenesis

We analyzed the genetic interactions of the *DISC1* and *dnlg1* by quantifying the morphological phenotypes in NMJ synaptic development (Figures 2A–H), then found that *DISC1^OE^* suppressed the synaptic bouton area more profoundly in *dnlg1^null^* heterozygous background with a synergetic interaction [Figure 2I: Two-way ANOVA (*F* (1, 52) = 4.30, *p* = 0.0431), Interaction of *DISC1^OE^* × *dnlg1^null^*] [Two-way ANOVA (*F* (1, 52) = 54.90, *p* < 0.0001), main effect of *DISC1^OE^*] [Two-way ANOVA (*F* (1, 52) = 25.43, *p* < 0.0001), main effect of *dnlg1^null^*] followed by Holm-Sidak’s multiple comparisons [+/+; *DISC1^OE^* (−) vs. +/+; *DISC1^OE^* (+), *p* = 0.0371, *t* = 2.140, *df* = 52] [*dnlg1^null^/+; DISC1^OE^* (−) vs. *dnlg1^null^/+*; *DISC1^OE^* (+), *p* < 0.0001, *t* = 4.940, *df* = 52] [+/+; *DISC1^OE^* (+) vs. *dnlg1^null^/+*; *DISC1^OE^* (+), *p* < 0.0001, *t* = 6.428, *df* = 52].

On the other hand, for the number of boutons, no significant difference was detected in the statistical interaction of *DISC1^OE^* × *dnlg1^null^* [Figure 2J: Two-way ANOVA (*F* (1, 52) = 0.4560, *p* = 0.5025), Interaction of *DISC1^OE^* × *dnlg1^null^*]. Therefore, *DISC1^OE^* and *dnlg1^null^* factors independently affected the number of boutons [Figure 2J: Two-way ANOVA (*F* (1, 52) = 7.739, *p* = 0.0075), main effect of *dnlg1^null^*] [Two-way ANOVA (*F* (1, 52) = 37.41, *p* < 0.0001), main effect of *DISC1^OE^*]. Accordingly, the Holm-Sidak’s multiple comparisons only within the same factor (fixing one factor) detected that the significant reduction in the number of boutons in *dnlg1^null^* heterozygous background [Figure 2J: *dnlg1^null^/+*; *DISC1^OE^* (−) vs. *dnlg1^null^/+*; *DISC1^OE^* (+), *p* = 0.0396, *t* = 2.40, *df* = 52] but no change in the +/+ wildtype background [+/+; *DISC1^OE^* (−) vs. +/+; *DISC1^OE^* (+), *p* = 0.1350, *t* = 1.518, *df* = 52].

Finally, regarding the number of axonal branch points, there was no significant statistical interaction of *DISC1^OE^* and *dnlg1^null^* [Figure 2K: Two-way ANOVA (*F* (1, 49) = 0.7244, *p* = 0.7244), Interaction of *DISC1^OE^* × *dnlg1^null^*]. Although the *dnlg1^null^* factor was detected as a significant source of variation [Two-way ANOVA (*F* (1, 49) = 7.528, *p* = 0.0085), main effect of *dnlg1^null^*] [Two-way ANOVA (*F* (1, 49) = 0.1258, *p* = 0.7244), main effect of *DISC1^OE^*], the Holm-Sidak’s multiple comparisons within the *dnlg1^null^* groups (fixing one factor) failed to detect any significant differences [*DISC1^OE^* (−); +/+ vs. *DISC1^OE^* (−); *dnlg1^null^/+*, *p* > 0.99, *t* = 0.00, *df* = 49] [*DISC1^OE^* (*+*); +/+ vs. *DISC1^OE^* (*+*); *dnlg1^null^/+*, *p* = 0.8611, *t* = 0.4886, *df* = 49], suggesting a specific suppression of the developmental stages of the synaptic bouton formation.

In summary, we have shown that the *dnlg1^null^* heterozygous mutation modifies the *DISC1^OE^* synaptic phenotype at the morphological level, with a synergistic suppression of total bouton area observed when both *DISC1^OE^* and *dnlg1^null^* heterozygous mutations are present. Overall, the NMJ anatomical results show that *DISC1* overexpression in the *dnlg1^null^* heterozygous background causes synaptic alterations at larval NMJs, potentially by convergent regulatory networks as discussed further in the next section.

## Discussion

Synaptic development and experience-dependent plasticity are widely viewed as convergent biological substrates across major mental disorders, including schizophrenia, since they integrate genetic risk with circuit maturation and cognitive phenotypes (Kirov et al., 2012; Fromer et al., 2014; Purcell et al., 2014; Hall et al., 2015). In this study, our data suggested a genetic interaction of *Neuroligin-1* with *DISC1* in glutamatergic synaptic development (Figure 2). Along with the other reported key molecules in the DISC1 network, we discuss the potential regulatory networks of DISC1 and Neuroligin in synaptic development and plasticity in the following sections.

Glutamatergic synaptogenesis has been considered as a complex assembly organization in which the trans-synaptic adhesion system constrains where pre- and postsynaptic specializations form, while intracellular scaffolds and signaling hubs determine how those nascent contacts stabilize, mature, and become plastic. Among the best-defined adhesion organizers are Neurexin-Neuroligin pairs, which can instruct presynaptic differentiation and align release sites with postsynaptic receptor/scaffold compartments (Südhof, 2008; Scheiffele et al., 2000; Krueger and Dean, 2012; Bemben et al., 2015). We previously found that the genetic interaction of *Neurexin* and *DISC1* in glutamatergic synaptogenesis, consistently using the *Drosophila* NMJ analysis (Pandey et al., 2017) (Supplementary Table S2). Our previous study showed that DISC1 exerts an antagonistic effect on Neurexin-dependent synapse formation, such that DISC1 suppresses synaptogenesis, whereas Neurexin promotes it (Pandey et al., 2017). In the present study, we demonstrated that DISC1 instead acts synergistically with Neuroligin, with both factors downregulating synapse formation (Figure 2 and Tables 1–3). These findings suggest a bidirectional regulatory relationship within the Neurexin/Neuroligin-DISC1 axis, whereby DISC1 opposes Neurexin-associated synaptogenic signaling while cooperating with Neuroligin to suppress postsynaptic maturation. Collectively, our data suggest that synapse formation is determined by the balance between presynaptic promotion and postsynaptic restraint, with the DISC1-Neuroligin complex acting as a postsynaptic brake on stabilization of immature synaptic contacts.

**Figure 3:**
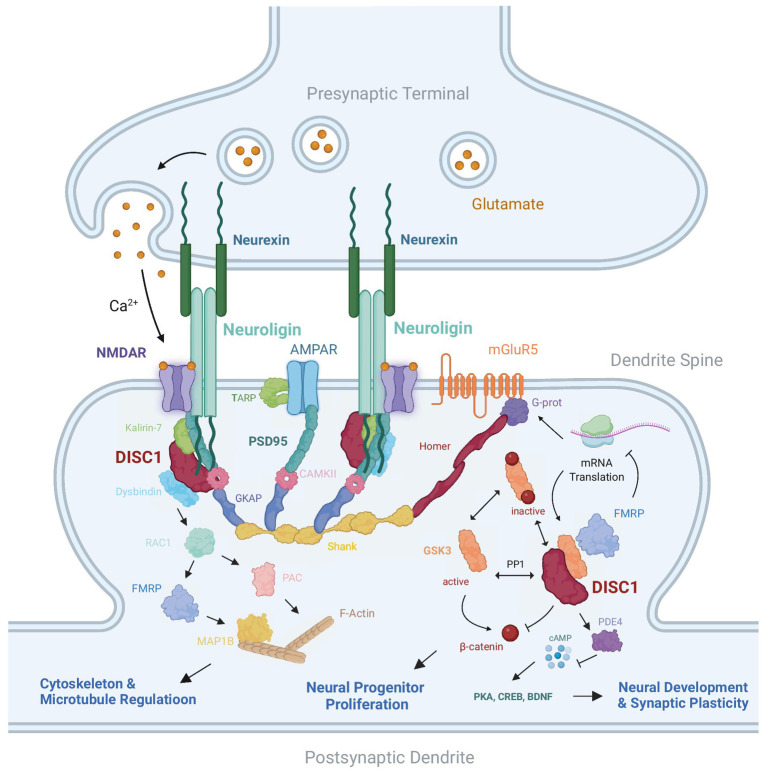
Putative schematic diagram of glutamatergic synaptogenesis integrating trans-synaptic adhesion, postsynaptic scaffold assembly, and DISC1-linked spine regulatory pathways. A presynaptic terminal (top) releases glutamate in a Ca^2+^-dependent manner into the synaptic cleft, where it engages postsynaptic receptors embedded in a dendritic spine on the postsynaptic dendrite (bottom). Across the cleft, presynaptic Neurexin and postsynaptic Neuroligin form an adhesion bridge that aligns pre- and postsynaptic specializations. On the postsynaptic side, Neuroligin couples to the excitatory scaffold PSD-95, linking adhesion to PSD assembly and receptor organization. This adhesion–scaffold axis coordinates the accumulation and stabilization of ionotropic glutamate receptors (NMDAR and AMPAR) and associated regulatory elements, including TARP, promoting synaptic receptor trapping and functional maturation. Within the PSD, multilayer scaffolding is represented by GKAP, Shank, and Homer. Downstream of mGluR5, G-protein (G-prot) signaling interfaces with local mRNA translation, regulated by FMRP, consistent with models in which group I mGluR signaling and translational control shape synaptic remodeling. The postsynaptic regulatory node centered on DISC1, linked to Kalirin-7 and downstream effectors that modulate spine structure and biochemical state, including PP1, GSK3 (inactive/active states indicated), β-catenin, PDE4, and cAMP. A proposed integrative pathway is emphasized wherein the Neurexin/Neuroligin complex interfaces functionally with a DISC1/Kalirin-7 module to influence spine morphogenesis and synapse stabilization, with RAC1 shown as a downstream small GTPase implicated in actin remodeling. Postsynaptic structural output is represented by F-actin remodeling and activity-linked kinase signaling (CaMKII) within the dendritic spine compartment. Additional DISC1-associated components depicted include Dysbindin and MAP1B, linking synaptic signaling to broader cytoskeleton & microtubule regulation, as well as schematic downstream outputs related to neural progenitor proliferation and neural development and synaptic plasticity, including a cAMP-linked effector axis labeled PAC and downstream transcriptional/signaling mediators such as PKA, CREB, and BDNF.

Combined with the previous literatures, our study can be integrated and putatively represented as in Figure 3, Neurexin-Neuroligin pairs and postsynaptic DISC1-Neuroligin complex potentially can be contributed as the central trans-synaptic organizer aligned with a downstream postsynaptic signaling/scaffold lattice (PSD95-GKAP-Shank-Homer) that interfaces with ionotropic glutamate receptors (NMDAR/AMPAR/TARP), metabotropic signaling (mGluR5/G-protein), local translation (FMRP/mRNA translation), and cytoskeletal remodeling (Kalirin-7/RAC1/F-actin; MAP1B).

A mechanistically persuasive route for DISC1-Neuroligin complex is the Kalirin-7/RAC1 pathway explicitly drawn adjacent to DISC1 (Figure 3, left pathway). DISC1 can modulate RAC1 activation dynamics by regulating Kalirin-7 access, shaping actin remodeling and spine morphology in response to glutamatergic signaling (Hayashi-Takagi et al., 2010). In parallel, our scheme has been proposed in which the Neurexin-Neuroligin pairs regulate a schizophrenia-relevant DISC1/Kalirin-7/RAC1 signalosome, potentially through a Kalirin-7-Neuroligin linkage that couples adhesion to cytoskeletal control (Owczarek et al., 2015). Together, our results in the *DISC1^OE^*; *dnlg1^null^/+* synaptic phenotypes (Figure 2) and the series of studies described above support an integrated postsynaptic interaction: Neuroligin-based synaptic adhesion may gate the spatial deployment of DISC1-Kalirin-7 signaling, thereby aligning trans-synaptic contact maturation with cytoskeletal remodeling required for stable spine formation (Figure 3) (Owczarek et al., 2015; Hayashi-Takagi et al., 2010).

Furthermore, a convergent model proposes that DISC1, Dysbindin, and FMRP intersect at RAC1-mediated control of actin and microtubules, shaping spine morphogenesis and synaptic maturation (Figure 3, left pathway) (Hayashi-Takagi et al., 2010; Castets et al., 2005; Furukubo-Tokunaga et al., 2016; Honda et al., 2024). Additionally, there is a DISC1-involved pathway (Figure 3, right pathway) that provides a developmental bridge through the GSK3/*β*-catenin module. RAC1 can modulate β-catenin nuclear accumulation in canonical Wnt signaling (Wu et al., 2008), and DISC1 has been linked to GSK3β/β-catenin stabilization and neuronal progenitor proliferation, connecting early developmental programs with later synaptic phenotypes (Mao et al., 2009; Singh et al., 2011).

Taken together, this study and the former research support a convergent, schizophrenia-relevant model in which Neuroligin-guided postsynaptic adhesion organizes the PSD environment and receptor alignment, together with the DISC1 network signaling that controls whether activity and developmental state are translated into stable synaptic structure and adaptive plasticity. The potential DISC1-Neuroligin interaction can therefore be considered as a key mechanistic advance: it points to a tractable integration node where adhesion-mediated synapse specification may recruit DISC1-involved modules that coordinate RAC1-dependent cytoskeletal remodeling, PSD composition control (TNIK), and cAMP-dependent plasticity state (PDE4/cAMP), all of which are implicated by genetic and neurobiological models of psychiatric risk (Fromer et al., 2014; Purcell et al., 2014; Wang et al., 2011; Millar et al., 2005; this study).

As limitations of this study, although we presented NMJ morphological phenotypes, we have not been able to demonstrate a direct functional interaction between DISC1 and Neuroligin at the protein level. Therefore, further studies, such as NMJ electrophysiology, calcium imaging, or active zone quantification using Bruchpilot staining, will help determine whether the morphological phenotypes result from their direct or indirect interactions, and whether DISC1 and Neuroligin function in the same pathway or multiple pathways involved in this process. An indirect supportive result from our previous study (Pandey et al., 2017) is that we examined active zone formation using Bruchpilot staining to analyze the functional interactions of DISC1 and Neurexin, the direct binding partner of Neuroligin. The result showed that DISC1 stimulates active zone density in a wild-type background but not in a *Neurexin* null background (Pandey et al., 2017), suggesting that Neurexin’s direct interactor, Neuroligin, may also be involved in this process.

Another notable limitation of the present study is that we did not directly capture interactions of the human endogenous DISC1 protein in the central nervous system, as we utilized a gain-of-function approach in the *Drosophila* model, focusing on peripheral NMJs. Although *Drosophila* NMJ models have contributed to understanding the molecular and genetic mechanisms of human diseases, cross-species verifications, including *Drosophila*, mice, and humans, will ultimately be critical to bridge the multilayered findings. Further studies, including a loss-of-function approach in mammalian models such as patient-derived organoids, are warranted in the future.

In addition, further investigations on the high-order behavioral phenotypes in *DISC1*^OE^; *dnlg1 ^null^/+* animals, such as social interactions (Samardžija et al., 2024), learning and memory (Honda et al., 2014, 2016; Honda, 2022), circadian rhythms (Lee et al., 2021), and sleep (Sawamura et al., 2008; Samardžija et al., 2024), will provide a holistic understanding of the DISC1-Neuroligin network at the systems level. By investigating genetic interactions in synaptic development *in vivo*, our study highlights a molecular mechanism regulated by *Neuroligin* and *DISC1* that may contribute to the understanding of the pathophysiology of schizophrenia.

## Data Availability

The original contributions presented in the study are included in the article/Supplementary material, further inquiries can be directed to the corresponding authors.

## Supplementary material

Supplementary Table S1 Normality tests for Figure 2 datasets.

Supplementary Table S2 Screening for potential DISC1 interactors

## Glossary

AMPAR: *α*-amino-3-hydroxy-5-methyl-4-isoxazolepropionic acid receptor
NMDAR: N-methyl-D-aspartate receptor
mGluR5: metabotropic glutamate receptor 5
TARP: transmembrane AMPAR regulatory protein
DISC1: Disrupted-in-Schizophrenia 1
PSD-95: postsynaptic density protein 95
GKAP: guanylate kinase–associated protein
Homer: Homer scaffolding protein
FMRP: fragile X mental retardation protein
PP1: protein phosphatase 1
GSK3: glycogen synthase kinase 3
PDE4: phosphodiesterase 4
cAMP: cyclic adenosine monophosphate
CaMKII: Ca^2+^/calmodulin-dependent protein kinase II
G-prot: heterotrimeric G-protein
RAC1: Ras-related C3 botulinum toxin substrate 1
MAP1B: microtubule-associated protein 1B
PKA: protein kinase A
CREB: cAMP response element–binding protein
BDNF: brain-derived neurotrophic factor
PAC: pituitary adenylate cyclase-activating polypeptide.

## References

[ref1] American Psychiatric Association (2022). Diagnostic and Statistical Manual of Mental Disorders: DSM-5-TR. 5th ed., text rev. Edn Washington, DC: American Psychiatric Association Publishing.

[ref2] Banovic D. Khorramshahi O. Owald D. Wichmann C. Riedt T. Fouquet W. . (2010). *Drosophila* neuroligin 1 promotes growth and postsynaptic differentiation at glutamatergic neuromuscular junctions. Neuron 66, 724–738. doi: 10.1016/j.neuron.2010.05.020, 20547130

[ref3] Bayat V. Jaiswal M. Bellen H. J. (2011). The BMP signaling pathway at the *Drosophila* neuromuscular junction and its links to neurodegenerative diseases. Curr. Opin. Neurobiol. 21, 182–188. doi: 10.1016/j.conb.2010.08.014, 20832291 PMC3095363

[ref1002] Bemben M. A. Shipman S. L. Nicoll R. A. Roche K. W. (2015). The cellular and molecular landscape of neuroligins. Trends Neurosci. 38, 496–505. doi: 10.1016/j.tins.2015.06.004, 26209464 PMC9381026

[ref4] Bradshaw N. J. Porteous D. J. (2012). DISC1-binding proteins in neural development, signalling and schizophrenia. Neuropharmacology 62, 1230–1241. doi: 10.1016/j.neuropharm.2010.12.027, 21195721 PMC3275753

[ref5] Brandon N. J. Sawa A. (2011). Linking neurodevelopmental and synaptic theories of mental illness through DISC1. Nat. Rev. Neurosci. 12, 707–722. doi: 10.1038/nrn3120, 22095064 PMC3954824

[ref6] Castets M. Schaeffer C. Bechara E. Schenck A. Khandjian E. W. Luche S. . (2005). FMRP interferes with the Rac1 pathway and controls actin dynamics in neuronal cells. Hum. Mol. Genet. 14, 835–844. doi: 10.1093/hmg/ddi077, 15703194

[ref7] Charng W.-L. Yamamoto S. Bellen H. J. (2014). Shared mechanisms between *Drosophila* peripheral nervous system development and human neurodegenerative diseases. Curr. Opin. Neurobiol. 27, 158–164. doi: 10.1016/j.conb.2014.03.001, 24762652 PMC4122633

[ref8] Collins C. A. DiAntonio A. (2007). Synaptic development: insights from *Drosophila*. Curr. Opin. Neurobiol. 17, 35–42. doi: 10.1016/j.conb.2007.01.001, 17229568

[ref10] Fromer M. Pocklington A. J. Kavanagh D. H. Williams H. J. Dwyer S. Gormley P. . (2014). De novo mutations in schizophrenia implicate synaptic networks. Nature 506, 179–184. doi: 10.1038/nature12929, 24463507 PMC4237002

[ref11] Furukubo-Tokunaga K. Kurita K. Honjo K. Pandey H. Ando T. Takayama K. . (2016). DISC1 causes associative memory and neurodevelopmental defects in fruit flies. Mol. Psychiatry 21, 1232–1243. doi: 10.1038/mp.2016.15, 26976042 PMC4993648

[ref12] Hall J. Trent S. Thomas K. L. O'Donovan M. C. Owen M. J. (2015). Genetic risk for schizophrenia: convergence on synaptic pathways involved in plasticity. Biol. Psychiatry 77, 52–58. doi: 10.1016/j.biopsych.2014.07.011, 25152434

[ref13] Hayashi-Takagi A. Takaki M. Graziane N. Seshadri S. Murdoch H. Dunlop A. J. . (2010). Disrupted-in-schizophrenia 1 (DISC1) regulates spines of the glutamate synapse via Rac1. Nat. Neurosci. 13, 327–332. doi: 10.1038/nn.2487, 20139976 PMC2846623

[ref14] Honda T. (2022). Optogenetic and thermogenetic manipulation of defined neural circuits and behaviors in *Drosophila*. Learn. Mem. 29, 100–109. doi: 10.1101/lm.053556.121, 35332066 PMC8973390

[ref15] Honda T. Kurita K. Arai Y. Pandey H. Sawa A. Furukubo-Tokunaga K. (2024). FMR1 genetically interacts with DISC1 to regulate glutamatergic synaptogenesis. Schizophrenia (Heidelb.) 10:112. doi: 10.1038/s41537-024-00532-7, 39604386 PMC11603133

[ref16] Honda T. Lee C. Y. Honjo K. Furukubo-Tokunaga K. (2016). Artificial induction of associative olfactory memory by optogenetic and thermogenetic activation of olfactory sensory neurons and octopaminergic neurons in *Drosophila* larvae. Front. Behav. Neurosci. 10:137. doi: 10.3389/fnbeh.2016.00137, 27445732 PMC4923186

[ref17] Honda T. Lee C. Y. Yoshida-Kashikawa M. Honjo K. Furukubo-Tokunaga K. (2014). Induction of associative olfactory memory by targeted activation of single olfactory neurons in *Drosophila* larvae. Sci. Rep. 4:4798. doi: 10.1038/srep04798, 24762789 PMC3999485

[ref18] Kirov G. Pocklington A. J. Holmans P. Ivanov D. Ikeda M. Ruderfer D. . (2012). *De novo* CNV analysis implicates specific abnormalities of postsynaptic signalling complexes in the pathogenesis of schizophrenia. Mol. Psychiatry 17, 142–153. doi: 10.1038/mp.2011.154, 22083728 PMC3603134

[ref19] Krueger D. D. Dean C. (2012). The role of neurexins and neuroligins in the formation, maturation, and function of vertebrate synapses. Curr. Opin. Neurobiol. 22, 412–422. doi: 10.1016/j.conb.2012.02.012, 22424845

[ref20] Kurusu M. Awasaki T. Masuda-Nakagawa L. M. Kawauchi H. Ito K. Furukubo-Tokunaga K. (2002). Embryonic and larval development of the *Drosophila* mushroom bodies: concentric layer subdivisions and the role of fasciclin II. Development 129, 409–419. doi: 10.1242/dev.129.2.409, 11807033

[ref21] Lee S. B. Park J. Kwak Y. Park Y. U. Nhung T. T. M. Suh B. K. . (2021). Disrupted-in-schizophrenia 1 enhances the quality of circadian rhythm by stabilizing BMAL1. Transl. Psychiatry 11:110. doi: 10.1038/s41398-021-01212-1, 33542182 PMC7862247

[ref22] Luo J. Li L. Niu M. Kong D. Jiang Y. Poudel S. . (2024). Genetic regulation of human brain proteome reveals proteins implicated in psychiatric disorders. Mol. Psychiatry 29, 3330–3343. doi: 10.1038/s41380-024-02576-8, 38724566 PMC11540848

[ref23] Mao Y. Ge X. Frank C. L. Madison J. M. Koehler A. N. Doud M. K. . (2009). Disrupted in schizophrenia 1 regulates neuronal progenitor proliferation via modulation of GSK3β/β-catenin signaling. Cell 136, 1017–1031. doi: 10.1016/j.cell.2008.12.044, 19303846 PMC2704382

[ref24] McCarthy S. E. Gillis J. Kramer M. Lihm J. Yoon S. Berstein Y. . (2014). De novo mutations in schizophrenia implicate chromatin remodeling and support a genetic overlap with autism and intellectual disability. Mol. Psychiatry 19, 652–658. doi: 10.1038/mp.2014.29, 24776741 PMC4031262

[ref25] Millar J. K. Pickard B. S. Mackie S. James R. Christie S. Buchanan S. R. . (2005). DISC1 and PDE4B are interacting genetic factors in schizophrenia that regulate cAMP signaling. Science 310, 1187–1191. doi: 10.1126/science.1112915, 16293762

[ref26] Moghaddam B. Javitt D. (2012). From revolution to evolution: the glutamate hypothesis of schizophrenia and its implication for treatment. Neuropsychopharmacology 37, 4–15. doi: 10.1038/npp.2011.181, 21956446 PMC3238069

[ref27] Mozer B. A. Sandstrom D. J. (2012). *Drosophila* neuroligin 1 regulates synaptic growth and function in response to activity and phosphoinositide-3-kinase. Mol. Cell. Neurosci. 51, 89–100. doi: 10.1016/j.mcn.2012.08.010, 22954894 PMC3494790

[ref28] Nagarkar-Jaiswal S. Lee P. T. Campbell M. E. Chen K. Anguiano-Zarate S. Gutierrez M. C. . (2015). A library of MiMICs allows tagging of genes and reversible, spatial and temporal knockdown of proteins in *Drosophila*. eLife 4:e05338. doi: 10.7554/eLife.05338, 25824290 PMC4379497

[ref29] O'Donnell K. H. Chen C. T. Wensink P. C. (1994). Insulating DNA directs ubiquitous transcription of the *Drosophila melanogaster* alpha 1-tubulin gene. Mol. Cell. Biol. 14, 6398–6408. doi: 10.1128/mcb.14.9.6398-6408.1994, 8065369 PMC359165

[ref30] Owczarek S. Bang M. L. Berezin V. Walmod P. S. (2015). Neurexin–neuroligin synaptic complex regulates DISC1/Kalirin-7/Rac1 signaling. Biomed. Res. Int. 2015:167308. doi: 10.1155/2015/167308, 26078884 PMC4452847

[ref31] Pandey H. Bourahmoune K. Honda T. Honjo K. Kurita K. Sato T. . (2017). Genetic interaction of DISC1 and Neurexin in the development of fruit fly glutamatergic synapses. NPJ Schizophr. 3:39. doi: 10.1038/s41537-017-0040-6, 29079805 PMC5660244

[ref32] Pardiñas A. F. Holmans P. Pocklington A. J. Escott-Price V. Ripke S. Carrera N. . (2018). Common schizophrenia alleles are enriched in mutation-intolerant genes and in regions under strong background selection. Nat. Genet. 50, 381–389. doi: 10.1038/s41588-018-0059-2, 29483656 PMC5918692

[ref33] Purcell S. M. Moran J. L. Fromer M. Ruderfer D. Solovieff N. Roussos P. . (2014). A polygenic burden of rare disruptive mutations in schizophrenia. Nature 506, 185–190. doi: 10.1038/nature12975, 24463508 PMC4136494

[ref34] Samardžija B. Petrović M. Zaharija B. Medija M. Meštrović A. Bradshaw N. J. . (2024). Transgenic *Drosophila melanogaster* carrying a human full-length DISC1 construct (UAS-hflDISC1) showing effects on social interaction networks. Curr. Issues Mol. Biol. 46, 8526–8549. doi: 10.3390/cimb46080502, 39194719 PMC11352338

[ref35] Sawamura N. Ando T. Maruyama Y. Fujimuro M. Mochizuki H. Honjo K. . (2008). Nuclear DISC1 regulates CRE-mediated gene transcription and sleep homeostasis in the fruit fly. Mol. Psychiatry 13:1069, 1138–1148. doi: 10.1038/mp.2008.101 18762802 PMC2727926

[ref36] Scheiffele P. Fan J. Choih J. Fetter R. Serafini T. (2000). Neuroligin expressed in nonneuronal cells triggers presynaptic development in contacting axons. Cell 101, 657–669. doi: 10.1016/S0092-8674(00)80877-6, 10892652

[ref37] Schizophrenia Working Group of the Psychiatric Genomics Consortium (2014). Biological insights from 108 schizophrenia-associated genetic loci. Nature 511, 421–427. doi: 10.1038/nature13595, 25056061 PMC4112379

[ref38] Singh K. K. De Rienzo G. Drane L. Mao Y. Flood Z. Madison J. . (2011). Common DISC1 polymorphisms disrupt Wnt/GSK3β signaling and brain development. Neuron 72, 545–558. doi: 10.1016/j.neuron.2011.09.030, 22099458 PMC3387684

[ref40] Südhof T. C. (2008). Neuroligins and neurexins link synaptic function to cognitive disease. Nature 455, 903–911. doi: 10.1038/nature07456, 18923512 PMC2673233

[ref41] Wang Q. Charych E. I. Pulito V. L. Lee J. B. Graziane N. M. Crozier R. A. . (2011). The psychiatric disease risk factors DISC1 and TNIK interact to regulate synapse composition and function. Mol. Psychiatry 16, 1006–1023. doi: 10.1038/mp.2010.87, 20838393 PMC3176992

[ref42] Wu X. Tu X. Joeng K. S. Hilton M. J. Williams D. A. Long F. (2008). Rac1 activation controls nuclear localization of β-catenin during canonical Wnt signaling. Cell 133, 340–353. doi: 10.1016/j.cell.2008.01.052, 18423204 PMC2390926

