# Supplementary material for: Genetic interaction of DISC1 and Neuroligin in regulation of glutamatergic synaptogenesis
Source: Front Neurosci. 2026 May 28;20:1832180. doi: 10.3389/fnins.2026.1832180 (PMC13253749; doi:10.3389/fnins.2026.1832180)
Supplement: Supplementary Table S1 — Normality tests for Figure 2 datasets. [file Table_1.pdf]

Normality Test — Figure 2I. Total Bouton Area

	DISC1 OE (-); dnlg1 null (-)	DISC1 OE (+); dnlg1 null (-)	DISC1(-); dnlg1 null (+)	DISC1 OE (+); dnlg1 null (+)
**Number of values**	19	11	13	14
**Minimum**	431.6	409.0	197.7	108.5
**25% Percentile**	483.3	419.0	234.6	125.5
**Median**	508.5	430.4	351.7	191.5
**75% Percentile**	558.0	469.0	486.0	287.7
**Maximum**	589.0	477.1	744.8	351.0
**Mean**	511.3	440.0	368.9	212.3
**Std. Deviation**	46.88	24.71	154.2	88.86
**Std. Error of Mean**	10.75	7.452	42.77	23.75
**Lower 95% CI of mean**	488.7	423.4	275.7	161.0
**Upper 95% CI of mean**	533.8	456.6	462.1	263.6
**Sum**	9714	4840	4795	2972
**D'Agostino & Pearson normality test**				
**K2**	1.184	2.558	5.343	3.129
**P value**	0.5534	0.2783	0.0691	0.2092
**Passed normality test (alpha=0.05)?**	Yes	Yes	Yes	Yes
**P value summary**	ns	ns	ns	ns
**Shapiro-Wilk normality test**				
**W**	0.9533	0.8802	0.8868	0.9004
**P value**	0.4495	0.1047	0.0883	0.1142
**Passed normality test (alpha=0.05)?**	Yes	Yes	Yes	Yes
**P value summary**	ns	ns	ns	ns

Normality Test — Figure 2J. Number of Boutons

	DISC1 OE (-); dnlg1 null (-)	DISC1 OE (+); dnlg1 null (-)	DISC1(-); dnlg1 null (+)	DISC1 OE (+); dnlg1 null (+)
**Number of values**	19	11	12	14
**Minimum**	85.00	94.00	75.00	45.00
**25% Percentile**	125.0	108.0	90.50	68.50
**Median**	146.0	131.0	108.5	88.50
**75% Percentile**	158.0	146.0	121.8	101.8
**Maximum**	180.0	158.0	134.0	113.0
**Mean**	140.6	127.5	106.8	85.36
**Std. Deviation**	26.27	21.87	18.24	21.56
**Std. Error of Mean**	6.026	6.594	5.267	5.762
**Lower 95% CI of mean**	128.0	112.9	95.24	72.91
**Upper 95% CI of mean**	153.3	142.2	118.4	97.81
**Sum**	2672	1403	1282	1195
**D'Agostino & Pearson normality test**				
**K2**	1.802	1.378	0.6690	1.630
**P value**	0.4061	0.5022	0.7157	0.4427
**Passed normality test (alpha=0.05)?**	Yes	Yes	Yes	Yes
**P value summary**	ns	ns	ns	ns
**Shapiro-Wilk normality test**				
**W**	0.9523	0.9510	0.9682	0.9187
**P value**	0.4323	0.6569	0.8912	0.2103
**Passed normality test (alpha=0.05)?**	Yes	Yes	Yes	Yes
**P value summary**	ns	ns	ns	ns

Normality Test — Figure 2K. Number of Branchpoints

	DISC1 OE (-); dnlg1 null (-)	DISC1 OE (+); dnlg1 null (-)	DISC1(-); dnlg1 null (+)	DISC1 OE (+); dnlg1 null (+)
**Number of values**	16	11	12	14
**Minimum**	11.00	8.000	13.00	7.000
**25% Percentile**	15.50	11.00	16.25	11.25
**Median**	17.00	12.00	17.50	15.00
**75% Percentile**	19.00	19.00	19.75	18.25
**Maximum**	26.00	24.00	21.00	23.00
**Mean**	17.75	14.09	17.75	14.93
**Std. Deviation**	3.941	5.147	2.417	4.999
**Std. Error of Mean**	0.9853	1.552	0.6977	1.336
**Lower 95% CI of mean**	15.65	10.63	16.21	12.04
**Upper 95% CI of mean**	19.85	17.55	19.29	17.82
**Sum**	284.0	155.0	213.0	209.0
**D'Agostino & Pearson normality test**				
**K2**	0.8702	2.017	0.4104	0.7601
**P value**	0.6472	0.3648	0.8145	0.6838
**Passed normality test (alpha=0.05)?**	Yes	Yes	Yes	Yes
**P value summary**	ns	ns	ns	ns
**Shapiro-Wilk normality test**				
**W**	0.9496	0.8723	0.9581	0.9527
**P value**	0.4835	0.0829	0.7569	0.6037
**Passed normality test (alpha=0.05)?**	Yes	Yes	Yes	Yes
**P value summary**	ns	ns	ns	ns
