# Supplementary material for: Genetic interaction of DISC1 and Neuroligin in regulation of glutamatergic synaptogenesis
Source: Front Neurosci. 2026 May 28;20:1832180. doi: 10.3389/fnins.2026.1832180 (PMC13253749; doi:10.3389/fnins.2026.1832180)
Supplement: Supplementary Table S2 — Screening for potential DISC1 interactors [file Table_2.pdf]

Supplementary Table S2: Screening for potential DISC1 interactors

Human schizophrenia risk factor genes and their fly homologue mutants under examination and reported

Risk Gene	HGNC ID	Fly Homologue	Fly Mutant Allele	Chromosome	Our Published Reports
ADAMTSL3	14633	CG31619	CG31619^{DG09212}	2	
Akt1	391	Akt1	Akt^{104226}	3	
ANK3	494	Ank2	Ank2^{f02001}	3	
CACNA1C	1390	Ca-a1D	Ca-α1D^{X10}	2	
CENTG2	16922	cenG1A	cenG1A^{EY01217}	2	
CNTNAP2	13830	Nrx-IV	Nrx-IV^{EY06647}	3	
CYFIP1	13759	CYFIP	Sra-1^{EY06562}	3	
DGCR8	2847	pasha	pasha^{EY01325}	3	
DLX1	2914	Dll	Dll^{md23}	2	
DTNBP1	17328	dysbindin	dysb^{e01028}	3	Furukubo-Tokunaga et al., 2016
ERBB4	3432	Egfr	Egfr^{f2}	2	
FAM69A	32213	CG12038	CG12038^{MB06619}	3	
FMR1	3775	Fmr1	Fmr1$^{\Delta 113M}$	3	Honda et al., 2024
Girdin	25523	Girdin	Girdin^{KG07727}	3	
GRIN1	4584	Nmdar1	Nmdar1^{DG23512}	3	
GRIN3A	16767	Ir85a	Ir85a^{MB04613}	3	
HTR2A	5293	5-HT2	5-HT2^{C1644}	3	
JAZF1	28917	CG12054	CG12054^{DG06105}	3	
KCNH2	6251	sei	sei^{HP21840}	2	
MAP2	6839	tau	tau^{MR22}	3	
MAPK3	6877	rl	rl^{10a}	2	
MDGA1	19267	ed	ed^{k01102}	2	
MTHFR	7436	CG7650	CG7650^{EY23633}	3	
MYO18B	18150	Mhcl	Mhcl^{NP1604}	3	
NDE1	17619	nudE	nudE^{G14350}	3	
NRG1	7997	vn	vn^{C221}	3	
NLGN1	14291	dnlg-1	dnlg1^{MI03763}	3	This study
NRXN1	8008	Nrx-1	dnrx1^{d08766}	2	Pandey et al., 2017
OLIG2	9398	tx	tx^{1}	3	
OPCML	8143	CG31646	CG31646^{MB09592}	2	
ORMDL3	16038	ORMDL	ORMDL^{e03591}	3	
PARK2	8607	park	park^{1}	3	
PLAA	9043	Plap	Plap^{d09025}	2	
PRSS16	9480	CG9953	CG9953^{KG09912}	3	
RGS4	10000	loco	loco^{KG02176}	3	
RPL5	10360	RpL5	RpL5^{2d2}	2	
SEMA3C	10725	Sema-2a	Sema-2a^{03021}	3	
SLC18A1	10934	Vmat	Vmat^{SH0459}	2	
SHOX	10853	CG34367	CG34367^{f00117}	2	
SRR	14398	CG8129	CG8129^{c04459}	3	
TBX6	11605	Dorsocross 1	Doc1^{MB02443}	3	
		Dorsocross 2	Doc2^{MB09116}	3	
TCF4	11634	da	da^{1}	2	
TPH1	12008	Trh	Trh^{c01440}	3	
TRAX	12380	Trax	Trax^{G18534}	3	

(Supplementary References)

1. Furukubo-Tokunaga, K., Kurita, K., Honjo, K., Pandey, H., Ando, T., et al., 2016. DISC1 causes associative memory and neurodevelopmental defects in fruit flies. *Molecular Psychiatry* 21, 1232–1243. https://doi.org/10.1038/mp.2016.15.
2. Honda, T., Kurita, K., Arai, Y., Pandey, H., Sawa, A., Furukubo-Tokunaga, K., 2024. FMR1 genetically interacts with DISC1 to regulate glutamatergic synaptogenesis. *Schizophrenia* 10(1), 112. https://doi.org/10.1038/s41537-024-00532-7.
3. Pandey, H., Bourahmoune, K., Honda, T., Honjo, K., Kurita, K., et al., 2017. Genetic interaction of DISC1 and Neurexin in the development of fruit fly glutamatergic synapses. *NPJ Schizophrenia* 3, 39. https://doi.org/10.1038/s41537-017-0040-6.
